# Evolution of amniote dentine apposition rates

**DOI:** 10.1098/rsbl.2022.0092

**Published:** 2022-04-27

**Authors:** Stephen P. Finch, Michael D. D'Emic

**Affiliations:** Department of Biology, Adelphi University, Garden City, NY 11530, USA

**Keywords:** dentine, Amniota, odontoblast

## Abstract

In amniotes, daily rates of dentine formation in non-ever-growing teeth range from less than 1 to over 25 μm per day. The latter value has been suggested to represent the upper limit of odontoblast activity in non-ever-growing teeth, a hypothesis supported by the lack of scaling between dentine apposition rates and body mass in Dinosauria. To determine the correlates and potential controls of dentine apposition rate, we assembled a dataset of apposition rates, metabolic rates and body masses for *ca* 80 amniote taxa of diverse ecologies and diets. We used phylogenetic regression to test for scaling relationships and reconstruct ancestral states of daily dentine apposition across Amniota. We find no relationship between body mass and daily dentine apposition rate (DDAR) for non-ever-growing teeth in Amniota as a whole or within major clades. Metabolic rate, the number of tooth generations, diet and habitat also do not predict or correspond with DDARs. Similar DDARs are found in large terrestrial mammals, dinosaurs and marine reptiles, whereas primates, cetaceans and some smaller marine reptiles independently evolved exceptionally slow rates. Life-history factors may explain the evolution of dentine apposition rates, which evolved rapidly at the origin of major clades.

## 1. Introduction

Palaeophysiological studies of extinct vertebrates are often limited in their explanatory power because growth data are more difficult to obtain than for extant species. One exception is the ubiquitously fossilized growth lines found in mineralized tissues such as teeth and bone, which follow regular time intervals of deposition [1]. In particular, teeth and associated tissues can serve as palaeophysiological archives at multiple temporal scales, which can inform fine-scale palaeoenvironmental and life-history reconstructions [1]. Key for reconstructing circadian and weekly patterns in growth is dentine—the tissue that makes up the bulk of a tooth's volume, which surrounds the pulp cavity and is overlain by enamel or cementum on the crown or root, respectively [2]. Dentine forms incrementally by apposition, produced by individual odontoblasts that leave behind processes that vary in morphology and density [3] and play a role in thermosensation [4]. Incremental lines of von Ebner are ubiquitous light/dark couplets that record daily apposition of dentine in teeth [2]. The number of incremental lines of von Ebner in a tooth indicates its formation time, whereas their thickness indicates the rate of dentine formation per day [1]. In some mammalian teeth, only annual growth increments are recorded (e.g. [2,5]), whereas the teeth of other taxa commonly preserve infradian lines in addition to daily ones [2,6]. Thus, careful histological study using multiple lines of evidence, explicit criteria for identification, and samples from more than one individual are preferred when inferring the periodicity of a given set of growth lines [7]. Once inferred, the daily dentine apposition rate (DDAR) can be compared across taxa to answer questions about life-history variation, palaeoecology and broad evolutionary patterns [1].

In amniotes, DDARs in non-ever-growing teeth range from less than 1 to over 25 μm per day [8]. The latter value has been explained as an approximate upper limit on the activity of odontoblasts in non-ever-growing teeth, a hypothesis supported by the lack of scaling between DDARs and body mass in Dinosauria [7,8]. However, scaling relationships and the hypothesized constraint on the DDAR in non-ever-growing teeth have yet to be empirically tested across Amniota. The broadest comparative studies of the DDAR have focused on a few derived amniote clades (e.g. Primates, [2] and Archosauria, [9]), but no study has analysed dentine growth across the entirety of Amniota, for which a rich dataset has been collected, often opportunistically over the past century with widely varying methodologies and standards of data reporting.

Herein, we present a collated and vetted dataset of published amniote DDAR values, augmented with novel histological sampling, in order to test the hypothesized daily limits of odontoblast activity, examine phylogenetic and allometric patterns of dentine growth evolution and reconstruct ancestral states of daily dentine apposition for major amniote clades through time.

## 2. Methods

We combined published and newly generated histological data to create a dataset of extinct and extant amniote taxa that spans approximately 255 Ma and several orders of magnitude in body mass. DDARs for over 125 amniote taxa were gathered from the literature; some of these were duplicate species or had measurement-related or other issues (see electronic supplementary material for details), yielding a final dataset of 80 taxa. Mean values were calculated when a range of values was reported for an individual tooth or taxon. The thicknesses of annual dentine increments in marine mammals (i.e. growth layer groups) and proboscideans were divided by 365 to obtain daily values. In studies where the DDAR was not reported, images from published figures were measured using ImageJ [10]. Only un-decalcified samples were included in our analysis; reported DDARs based on decalcified thin sections were not included due to known issues with tissue shrinkage [11]. We excluded samples from embryonic individuals.

To broaden our literature-based dataset, we thin-sectioned one isolated maxillary or premaxillary tooth of the sauropod dinosaur *Abydosaurus* (DNM 16–20), one isolated dentary tooth of the ornithopod dinosaur *Tenontosaurus* (OMNH 08137), two canines (YPM 16131 & UM 118419) and one premolar (YPM 14723) of the pantodont mammal *Coryphodon* according to standard palaeohistological techniques [12]. Specimens were embedded in epoxy resin, bisected with a low-speed diamond blade saw in either the mesiodistal or transverse plane, ground with 600 grit sandpaper, mounted to frosted glass slides with cyanoacrylate, cut to *ca* 0.5 mm thickness and sanded again using 600 and then 1200 grit sandpaper to a thickness of *ca* 100 μm. Montages of thin sections were created from images taken at 50× or 200× total magnification using a Zeiss Axioimager Z2 system running Zen2 software. We traced von Ebner incremental lines on these stitched images using Adobe Illustrator and measured their thicknesses using ImageJ v. 1.53a [10]. High-resolution montaged micrographs are available on Morphobank at project no. P4216. We followed methods and arguments laid out in D'Emic *et al*. [7] in identifying von Ebner incremental width in *Coryphodon*, *Abydosaurus* and *Tenontosaurus* as daily rather than infradian or higher order.

We assembled a composite phylogeny for the specimens in our dataset based on several recent studies (see electronic supplementary material for sources). We then plotted DDAR on this composite phylogeny using the phytools package in R [13]. Branch lengths were estimated based on geological ages, which were taken from the Paleobiology Database and assigned to our tree using the R code provided by Graeme Lloyd (http://www.graemetlloyd.com/methdpf.html).

Adult body mass estimates for extinct taxa were gathered from published estimates or estimated from reliable proxies such as stylopodial circumference (e.g. [14]) or cranial width [15]. Ichthyosaur and mosasauroid body masses were calculated using a length–mass equation developed for cetaceans [16], since they have similar body plans. For extant taxa, body masses were gathered from databases AnAge and Animal Diversity Web. When available, male and female values were averaged. For four taxonomically indeterminate specimens, the mean body mass of all known members of the smallest clade that the specimen could be assigned to was used to represent the taxon (see electronic supplementary material). For *Priosphenodon avelasi*, body mass was estimated using similarly sized and proportioned extant varanids as a proxy. We gathered resting metabolic rates from the literature and converted measurements into watts following Grady *et al*. [17].

We tested for the influence of diet, di- versus polyphyodonty and habitat using *t*-tests and ANOVA in PAST4 [18]. We regressed natural log-transformed mean DDARs on natural log-transformed mean body mass, both with and without accounting for phylogenetic influence using phylogenetic generalized least-squares and ordinary least-squares regression, respectively, following the methods and using the R code provided in D'Emic *et al*. [7]. A *p*-value threshold of 0.006 was applied using a Bonferroni correction. We then plotted a phylomorphospace of these variables using phytools [13]. All R code, raw data, phylogenetic trees and branch lengths are provided in the electronic supplementary material.

## 3. Results

**Figure 1.**
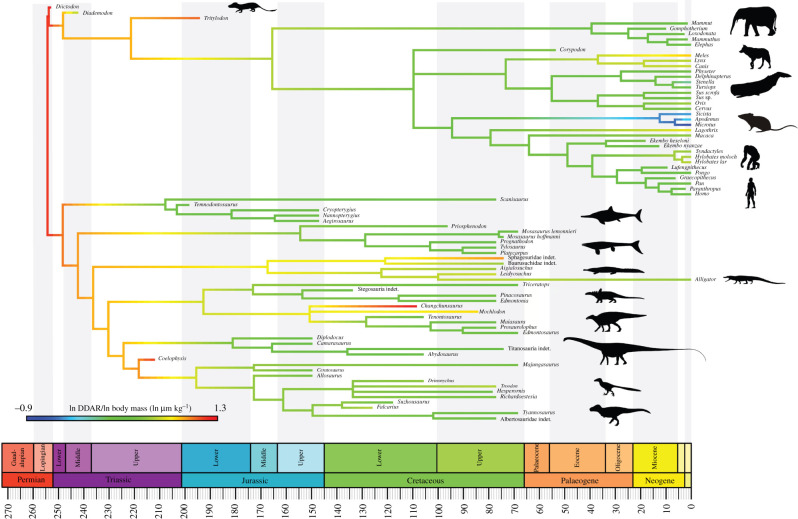
Body mass-corrected DDARs plotted on phylogeny and scaled to geological time. Warmer colours indicate higher daily apposition rates and cooler colours indicate lower rates per unit body mass. Silhouettes from phylopic.org; see acknowledgements for credits.

**Figure 2.**
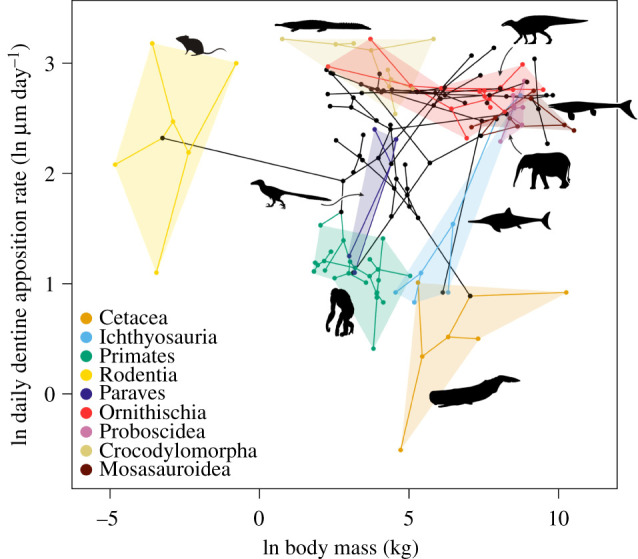
Phylomorphospace of body mass versus DDAR within Amniota. Nine derived clades are highlighted. Silhouettes from phylopic.org; see acknowledgements for credits.

We reconstruct the ancestral amniote DDAR as relatively high (*ca* 18 μm day^–1^; electronic supplementary material, table S1), with later decreases in mammals and several reptile groups (see Discussion below). Overall, there is an extremely weak but statistically significant decrease in DDAR per unit body mass through time (*r*^2^ = 0.06; *p* = 0.03). When DDAR is divided by body mass, few outliers are found—rodents have exceptionally high DDAR for their body mass, whereas some non-mammalian therapsids and archosaurs have exceptionally low DDAR per unit body mass (figures 1 and 2). Little overlap exists in the body mass–DDAR morphospace. Rodents, paravians and ichthyosaurs have a broad range of DDAR, whereas dinosaurs, crocodyliforms, mosasauroids and proboscideans have a narrow range (figure 2). Part of the morphospace is unoccupied by our dataset—we found no taxa that are both smaller than and have slower DDAR than the average primate (figure 2).

There is no significant relationship between DDAR and body mass across Amniota found with OLS regression (*n* = 80; *p* ∼ 0.03; *r*^2^ ∼ 0.06; note Bonferroni-corrected *p*-value threshold = 0.006; electronic supplementary material, table S2) or PGLS regression (Pagel's lambda approximately 1), demonstrating no meaningful relationship between body mass and DDAR. Furthermore, OLS and PGLS regressions detect no significant relationship within any major clade within Amniota (electronic supplementary material, table S2). There is likewise no relationship between resting metabolic rate and DDAR (*p* = 0.99; *r* < 0.01; electronic supplementary material, figure S3).

There is a trend of thinner DDAR per unit of body mass in some semiaquatic or aquatic animals versus terrestrial animals, though no overall difference in mean in these two groups (*t*-test; *p* = 0.42; electronic supplementary material, figure S1). By contrast, the median and variance are substantially different between terrestrial versus semiaquatic/aquatic taxa (*F*-test; *p* < 1 × 10^−8^; Mann–Whitney *U* test; *p* = 0.004). Some derived aquatic groups have very thin incremental lines (cetaceans, mosasaurs, ichthyosaurs and plesiosaurs), though when body mass is accounted for, they do not stand out from the amniote mean. DDAR does not differ among dietary categories (ANOVA: *F* = 0.028; *p* = 0.97; Kruskal–Wallis *p* = 0.81; electronic supplementary material, figure S4). Diphyodont animals have slightly lower DDAR than polyphyodont animals (*t*-test; *p* = 0.04; electronic supplementary material, figure S5).

The earliest archosaurs largely retained the ancestral condition of relatively high DDAR (*ca* 16 μm day^–1^), with no substantial change at the origin of Crocodylomorpha, Dinosauria, Ornithischia or Saurischia. Within Archosauria, some Mesozoic crocodyliforms appear to have evolved exceptionally high DDARs, whereas paravians evolved very low rates. Within Mammalia, the ancestral DDAR is modest (*ca* 11 μm), whereas Cetacea and Primates evolved much lower rates (*ca* 2 and 5 μm, respectively).

## 4. Discussion

Our greatly expanded dataset supports previous work reporting no significant relationship between DDAR and body mass in Dinosauria or Archosauria [7,8], showing that this lack of relationship extends to several major amniote clades and to Amniota as a whole. Indeed, the lowest DDARs are found in some of the largest animals in the dataset (whales), whereas the highest DDARs are found in some of the smallest (rodents and smaller crocodyliforms). The reconstructed high DDARs near the base of Amniota are concordant with the large values in some amniote outgroup taxa, including *Pholiderpeton attheyi* (approx. 21 µm as measured from fig. 9C in [19]) and an indeterminate dissorophoid temnospondyl (approx. 24 µm as measured from fig. 2A in [20]). Further sampling of non-amniotes is needed to better constrain the ancestral amniote condition.

The hypothesized upper physiological limit on odontoblast activity [8] is supported. Additionally, a lower limit was observed for smaller amniotes: no taxon in our dataset smaller than *ca* 10 kg had a DDAR lower than approximately 3 μm day^–1^. This may indicate a lower limit to odontoblastic activity in these lineages due to metabolic constraints imposed by the surface-area-to-volume ratio in very small cells (e.g. [21]).

Like body mass, metabolic rate, diet, habitat and di- versus polyphyodonty do not predict DDAR values in Amniota (electronic supplementary material), suggesting that the explanation for derived DDARs in various clades lies in their peculiar life histories. In mammals, the DDAR and other measurements of the rate of tooth development have been explained in the context of life-history evolution (see Hogg [1] and references therein) and the pace of hormone-regulated biological rhythms [22]. Evolutionary shifts in these rhythms, extended parental care, prolonged gestation times, changes in developmental rate and/or dietary shifts to softer foodstuffs could allow for the evolution of lower DDAR. We hypothesize that this mammalian/life-history explanation extends to other clades with derived DDAR, such as ichthyosaurs or paravians. To test this hypothesis, future work should sample bones and teeth of the same individuals of these clades, as well as expand our sample to lineages with rapidly forming teeth (e.g. bovids, equids and castorids) and small body size (e.g. small squamates, varanids and chiropterans). Similarly, resampling and/or restudy of the decalcified and/or ambiguously reported specimens already reported in the literature would increase the size of the dataset by over 60%, while not impacting any specimens that had not already been destructively sampled. The dataset and analysis herein provide a framework of expected DDAR values for various clades, facilitating the interpretation of the periodicity of incremental lines in the dentine of extinct animals. Richer sampling will lead to a better understanding of the factors that relate to DDAR and with it more powerful models of palaeophysiological reconstruction.

## Data Availability

The datasets supporting this article have been uploaded as part of the electronic supplementary material [23].
